# Angiopoietin 2 Alters Pancreatic Vascularization in Diabetic Conditions

**DOI:** 10.1371/journal.pone.0029438

**Published:** 2012-01-17

**Authors:** Sophie Calderari, Cécile Chougnet, Maud Clemessy, Hervé Kempf, Pierre Corvol, Etienne Larger

**Affiliations:** 1 INSERM U833, Collège de France, Paris, France; 2 Laboratoire de Physiopathologie, Pharmacologie et Ingénierie Articulaires UMR 7561, CNRS-Nancy-Université, Vandoeuvre-Lès-Nancy, France; 3 Faculté de Médecine, Université Paris Descartes, Paris, France; University of Bremen, Germany

## Abstract

**Aims/hypothesis:**

Islet vascularization, by controlling beta-cell mass expansion in response to increased insulin demand, is implicated in the progression to glucose intolerance and type 2 diabetes. We investigated how hyperglycaemia impairs expansion and differentiation of the growing pancreas. We have grafted xenogenic (avian) embryonic pancreas in severe combined immuno-deficient (SCID) mouse and analyzed endocrine and endothelial development in hyperglycaemic compared to normoglycaemic conditions.

**Methods:**

14 dpi chicken pancreases were grafted under the kidney capsule of normoglycaemic or hyperglycaemic, streptozotocin-induced, SCID mice and analyzed two weeks later. Vascularization was analyzed both quantitatively and qualitatively using either in situ hybridization with both mouse- and chick-specific RNA probes for VEGFR2 or immunohistochemistry with an antibody to nestin, a marker of endothelial cells that is specific for murine cells. To inhibit angiopoietin 2 (Ang2), SCID mice were treated with 4 mg/kg IP L1–10 twice/week.

**Results:**

In normoglycaemic condition, chicken-derived endocrine and exocrine cells developed well and intragraft vessels were lined with mouse endothelial cells. When pancreases were grafted in hyperglycaemic mice, growth and differentiation of the graft were altered and we observed endothelial discontinuities, large blood-filled spaces. Vessel density was decreased. These major vascular anomalies were associated with strong over-expression of chick-Ang2. To explore the possibility that Ang2 over-expression could be a key step in vascular disorganization induced by hyperglycaemia, we treated mice with L1–10, an Ang-2 specific inhibitor. Inhibition of Ang2 improved vascularization and beta-cell density.

**Conclusions:**

This work highlighted an important role of Ang2 in pancreatic vascular defects induced by hyperglycaemia.

## Introduction

Insulin-producing beta-cells and endothelial cells in the pancreatic islets of Langerhans exchange bidirectional signals that are necessary for development, differentiation and proper function of both endocrine and vascular compartments [1]. Various beta-cell-secreted angiogenic factors, like vascular endothelial growth factor (VEGF) [2], [3], [4], are crucial for maintaining a dense and fenestrated capillary network that affords proper insulin secretion [5]. With regards to VEGF receptors, while vessels of the exocrine tissue express the inactive (VEGF-R1) isoform, islet endothelial cells express the active (VEGFR2) isoform [2], [6]. Beta-cells produce several other pro-angiogenic but also anti-angiogenic factors, like angiopoietin-1 (Ang1) and thrombospondin-1 (Tsp1), respectively. While Ang1-deficient mice are non-viable, due to severe vascular defects [7], Tsp1-null mice have large and highly vascularized islets [8]. Mutant mice lacking the two insulin genes also exhibit increased pancreatic vascularization without change in VEGF and VEGFR2 expression [9]. Conversely, islet endothelial cells act on endocrine cells. During early pancreatic development, vascular endothelial cells are key inducers for islet differentiation [10] and, endothelial cell signals, such as those involved in matrix-integrin interaction, modulate beta-cell proliferation and function [11], [12].

In situations such as pregnancy, postnatal development, obesity or insulin resistance, islet mass adapts to increased insulin demand [13]. During pregnancy, islet endothelial-cell secreted hepatocyte growth factor stimulates beta-cell proliferation by downregulating Tsp1 [14]. In type 2 diabetes, the possibility that hyperglycaemia itself further affects beta-cell mass via islet endothelial cell alterations received little attention until now [1]. However, alterations of vasculature exist in several type 2 diabetes animal models. The db/db mouse shows decreased capillary density and, increase in the mean and diversity of capillary size, associated with pericapillary oedema, fibrosis and irregularity of the endothelial luminal surface [15], [16]. Likewise spontaneously (nonobese) diabetic Goto-Kakizaki rats have deficient islet vascularization from neonatal life to adulthood [17]. GK rats show progressively signs of islet endothelial activation, inflammation, vessel alterations, fibrosis and of beta-cell loss [18]. Islet endothelium alterations may be early events in the pathogenesis of hyperglycaemia as they have also been observed in both prediabetic (nonobese) Torii and Zucker diabetic fatty (ZDF) rats [19], [20]. In intrauterine growth restriction animals, which are prone to insulin resistance, obesity, and type 2 diabetes, the reduction of islet vascular density precedes that of beta-cell mass by several weeks [21]. Neonatal exendin-4 treatment of these rats normalizes islet vascular density, by increasing VEGF protein and prevents beta-cell mass deterioration and diabetes onset [21], [22].

Therefore, islet vascularization appears to be a key element in the control of beta-cell mass expansion to increased insulin demand [1]. Here, we investigated how hyperglycaemia impairs expansion and differentiation of the growing pancreas, using the xenogenic (avian) embryonic pancreas grafting under Severe Combined Immuno-Deficient (SCID) mouse kidney capsule. Avian models have been useful in morphogenesis and organogenesis studies [23] and chick pancreas developmental biology shares many similarities with that of mammals [24], [25]. Grafting of embryonic chick pancreas in SCID mice allowed us to characterize the avian or murine origin (pancreatic or vascular) of growth signals and to identify and differentially modulate some of them, to dissect their role during hyperglycaemia.

## Results

### Pancreatic chimeras consisting of chicken-derived endocrine and exocrine cells and vessels with endothelial cells of murine origin

**Figure 1:**
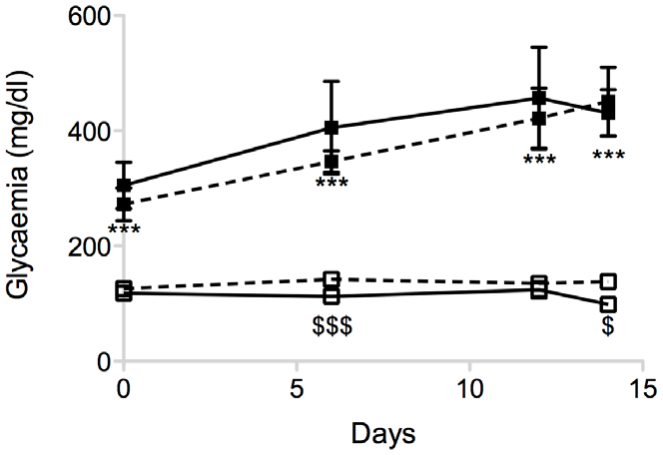
Blood glucose concentrations of SCID mice during the 2 weeks post-graft of chicken pancreas. Continious line, white square: control mice, n = 12. Continious line, black square: STZ mice, n = 7. Dotted line, white square: L1–10 treated control mice, n = 5. Dotted line, black square: L1–10 treated STZ mice, n = 5. Mice were used for grafting experiments between 3 to 5 days after STZ or citrate buffer injections. *** p<0.001 control and L1–10 treated control mice vs STZ and L1–10 treated STZ mice. $ p<0.05, $$$ p<0.001 control mice vs: L1–10 treated control mice.

**Figure 2:**
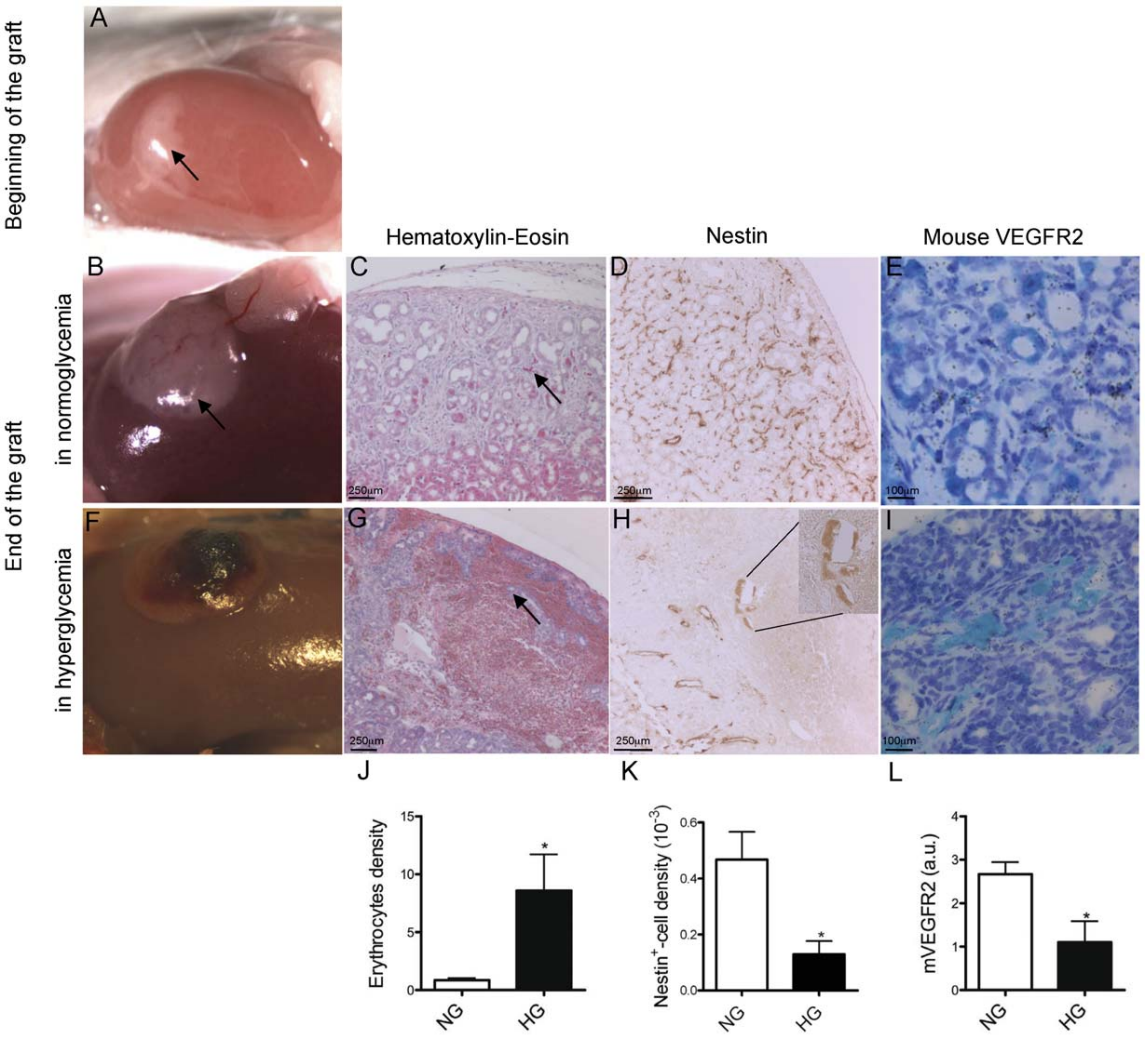
Graft of embryonic chick pancreas under SCID mouse kidney capsule. At 14 dpi embryonic chick pancreas (A) was grafted under the kidney capsule of a normoglycaemic SCID mouse and analyzed 2 weeks post-transplantation (B). At that time, hematoxylin-eosin coloration (C) enabled us to observe many vessels filled with erythrocytes (arrow). Immunohistochemistry for nestin (D) and *in situ* hybridization for mouse VEGFR2 probe (E) indicated the murine origin of endothelial cells in the pancreatic graft. In hyperglycaemic conditions (F–I), dark spots were present on the top of the pancreatic grafts (F). Vascularization was disorganized with large blood-fill spaces (G, arrow) associated with vessel discontinuities (H, insert). Morphometry analyses of erythrocytes staining (G), nestin immunohistochemistry (H) and mouse VEGFR2 *in situ* hybridization (I) showed a decreased number of endothelial cells (J, K and L). Student *t-*test *p<0.05, n = 4–6.

**Figure 3:**
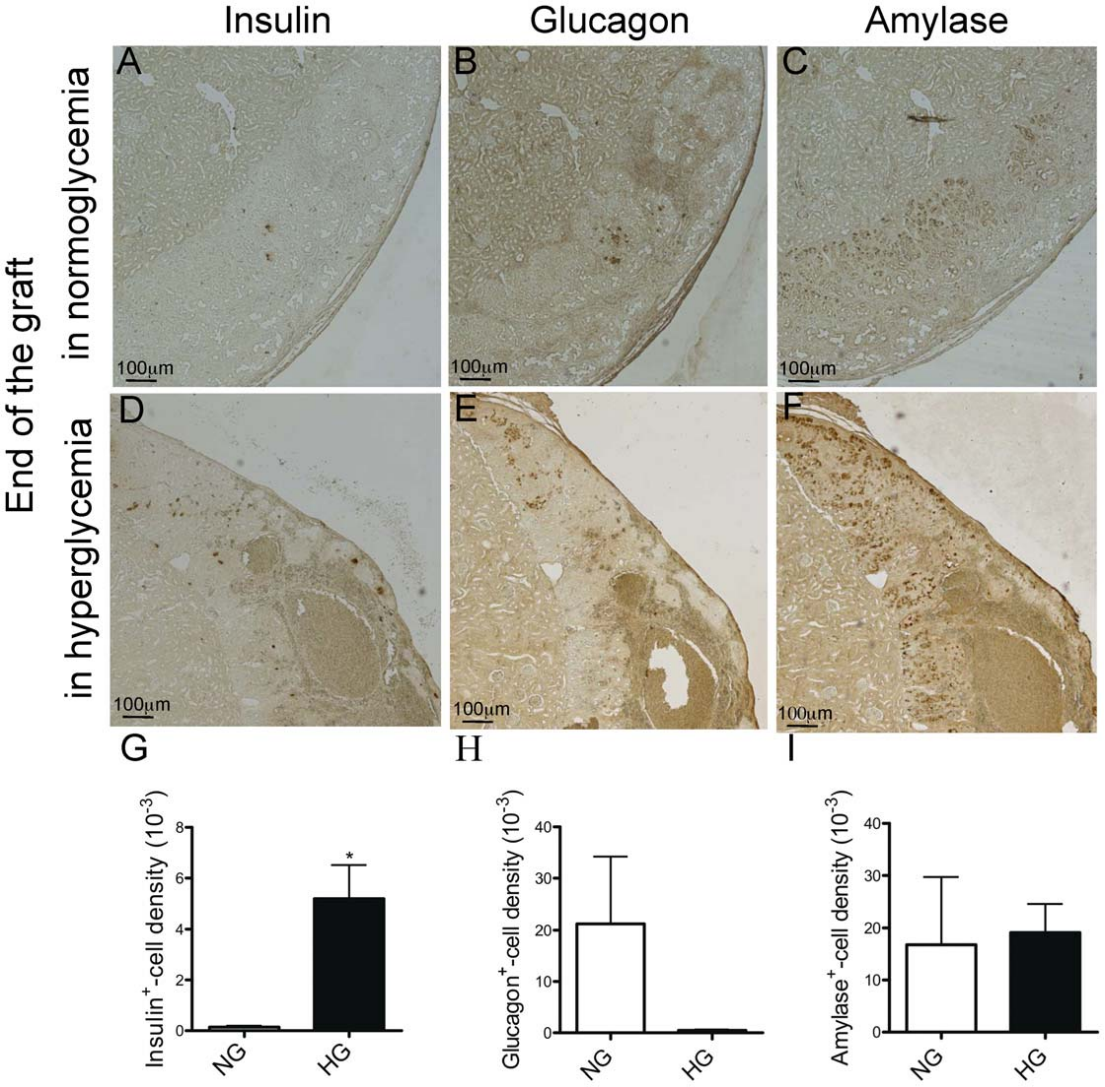
Endocrine and exocrine differentiation in normo and hyperglycaemic pancreas. Insulin (A, D), glucagon (B, E) and amylase (C, F) positive-cells in 2 weeks grafted pancreata in normoglycaemic (A–C) and hyperglycaemic conditions (D–F). Quantification of insulin (G), glucagon (H) and amylase (I) positive-cells density. Hyperglycaemia increased beta-cell density (G). Student *t-* test *p<0.05, n = 3–5.

In embryonic chick pancreas at 14 dpi before graft, we detected few endocrine cells using anti-insulin and anti-glucagon antibodies [26] and rare exocrine cells using an anti-amylase antibody (data not shown). Fourteen-dpi chick pancreas were grafted under the kidney capsule of normoglycaemic SCID mice (Fig. 1) and analyzed 2 weeks later (Fig. 2.A and B). Under normoglycaemia, the average size of the transplanted chick pancreas had increased by 3.8 fold as compared to 14 dpi pancreas (75±32 mm^2^ before, n = 3 and 287±27 mm^2^ after, n = 4, p<0.01). Endocrine and exocrine differentiation was maintained (Fig. 3.A–C). Grafts were well vascularized as observed macroscopically (Fig. 2.B) and microscopically (Fig. 2.C–E). Vessels were filled with erythrocytes, confirming their functionality (Fig. 2.C). The use of species-specific probes for immunohistochemistry (Fig. 2.D) or *in situ* hybridization (Fig. 2.E) led to characterize the origin of the graft vascularization. *In situ* hybridization revealed the expression of c-VEGFR2 in 14 dpi chick pancreas before graft but not in 2 weeks grafts (data not shown. In contrast m-VEGFR2 that was not detected in pancreatic vessels before graft was clearly expressed on most vessels 2 weeks after graft (n = 6, Fig. 2.E). Nestin, a marker for mouse endothelial cell, never detected endothelial cells of avian origin by immunohistochemistry (data not shown), confirmed the murine origin of proliferative vessels within grafts (n = 4, Fig. 2.D). The pattern of nestin staining observed in pancreatic graft was similar to that of Von Willebrand Factor and CD31 staining (data not shown). We have thus created chimeras consisting of chicken-derived endocrine and exocrine cells and vessels with endothelial cells of murine origin.

### Hyperglycaemia induced pancreatic vascular anomalies and increased angiopoietin 2

Hyperglycaemia was induced by a single STZ injection to SCID mice. During the two weeks of grafts, STZ injected SCID mice were hyperglycaemic as compared to citrate buffer control SCID mice (Fig. 1). We did not detect effect of the graft on blood glucose levels of recipients.

Effect of hyperglycaemia on angiogenesis was examined 2 weeks after graft of 14 dpi chick pancreas under the kidney capsule of diabetic SCID mice. The average pancreas size was not altered by hyperglycaemia (287±27 mm^2^, control, n = 4; 384±54 mm^2^, hyperglycaemic, n = 5). However, hyperglycaemia increased beta-cell density by 35 fold, as compared to normoglycaemia (Fig. 3.D). Pancreatic vasculature was overtly disorganized. All grafts exhibit macroscopic haemorrhages (Fig. 2.F). At the microscopic level, these haemorrhages were observed as large blood-filled spaces (Fig. 2.G). Many of these pancreatic vascular structures were only partly surrounded by endothelial cells, (Fig. 2.H). We thus quantified the vascular compartment by assessment of erythrocyte area and found that hyperglycaemia increased the total area of vascular compartment by 9 fold (Fig. 2.J). By contrast, endothelial cell density was decreased by hyperglycemia, as assessed after either nestin immunohistochemistry (Fig. 2.H and K) or *in situ* hybridization with the mouse-specific VEGFR2 probe (Fig. 2.I and L).

**Figure 4:**
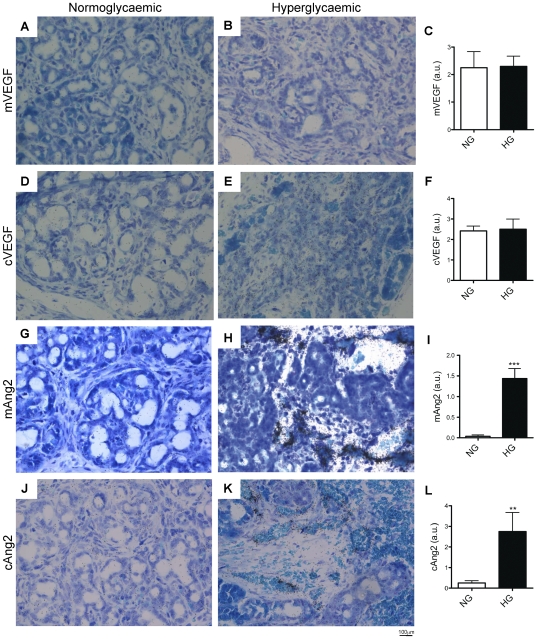
*In situ* hybridization of angiogenic factors in pancreatic grafts after 2 weeks hyperglycaemia. Expression of mouse VEGF (A, B), chick VEGF (D, E), mouse Ang2 (G, H) and chick Ang2 (J, K) probes in normoglycemic (A, D, G, J) and hyperglycaemic grafts (B, E, H, K) was analyzed by *in situ* hybridization. C, F, I and L, semi-quantification of labeling intensity between normoglycaemic and hyperglycaemic conditions. Ang2 expressions were increased by hyperglycaemia. Student *t*-test, **p<0.01, ***p<0.001, n = 5.

Abnormal expression of pro-angiogenic factors (VEGF, Ang1) or anti-angiogenic factors (Tsp1, Ang2) might trigger these hyperglycaemia-induced vessel alterations. *In situ* hybridization, hardly detected expression of mVEGF, cVEGF, Tsp1, mAng2 and cAng2 in normoglycaemic grafts (Fig. 4.A, D, G and J). While hyperglycaemic condition did not alter mVEGF (Fig. 4.B and C), cVEGF (Fig. 4.E and F) and Tsp1 expression (data not shown), hyperglycaemia increased mAng2 (Fig. 4.H and I) and cAng2 expression (Fig. 4.K and L). We next investigated the respective roles of VEGF and Ang2, in hyperglycaemia-induced alterations of angiogenesis.

### Over-expression of the pro-angiogenic factor VEGF did not counteract hyperglycaemic induced vascular defects

A major advantage of chick tissues is the susceptibility to avian-specific retroviruses. Replication-competent ASLV long terminal repeat with a Splice acceptor (RCAS) vectors are powerful tools to introduce and over-express genes specifically in avian tissues [27].

**Figure 5:**
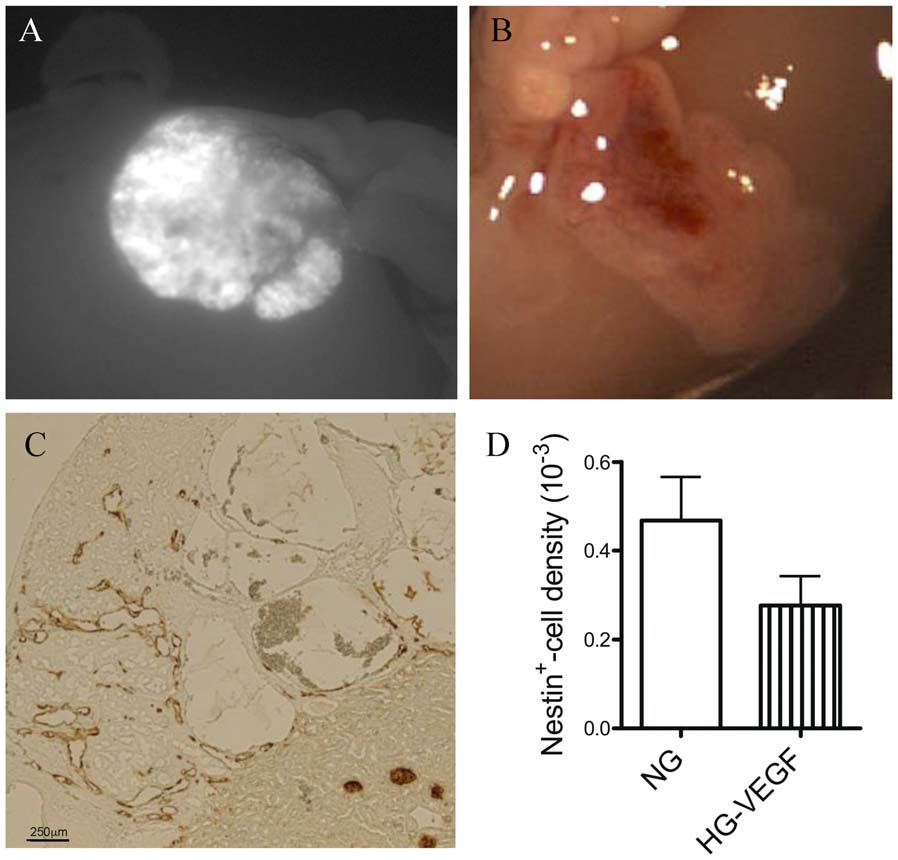
Over-expression of VEGF using RCAS retrovirus. Embryonic chick pancreas was infected with RCAS-GFP just before grafting. GFP was only and strongly expressed in the graft (A). In B, a photograph of a RCAS-VEGF-infected and grafted pancreas after 2 weeks hyperglycaemia showed the presence of blood filled spaces. In C, nestin immunohistochemsitry in VEGF-over-expressing and grafted pancreas after 2 weeks in hyperglycaemic conditions and in D, morphometry analyses of nestin immunohistochemistry (n = 4).

The first approach was to specifically over-express VEGF in pancreatic grafts, using an avian-specific retrovirus RCAS carrying the gene encoding the VEGF (RCAS-VEGF). Pancreata were infected *in vitro* just before grafting. VEGF over-expression did not improve significantly pancreatic angiogenesis in hyperglycaemic SCID mice, as evidenced by nestin staining quantification (Fig. 5.C and D). The vasculopathy pattern of these grafts was similar to that of untreated pancreata. Because VEGF over-expression did not decrease Ang2 expression in hyperglycaemic conditions (data not shown), we hypothesized that Ang2 over-expression may be directly involved in these vessels defects.

### Inhibition of Ang2 improved vascular anomalies in hyperglycaemic pancreas

**Figure 6:**
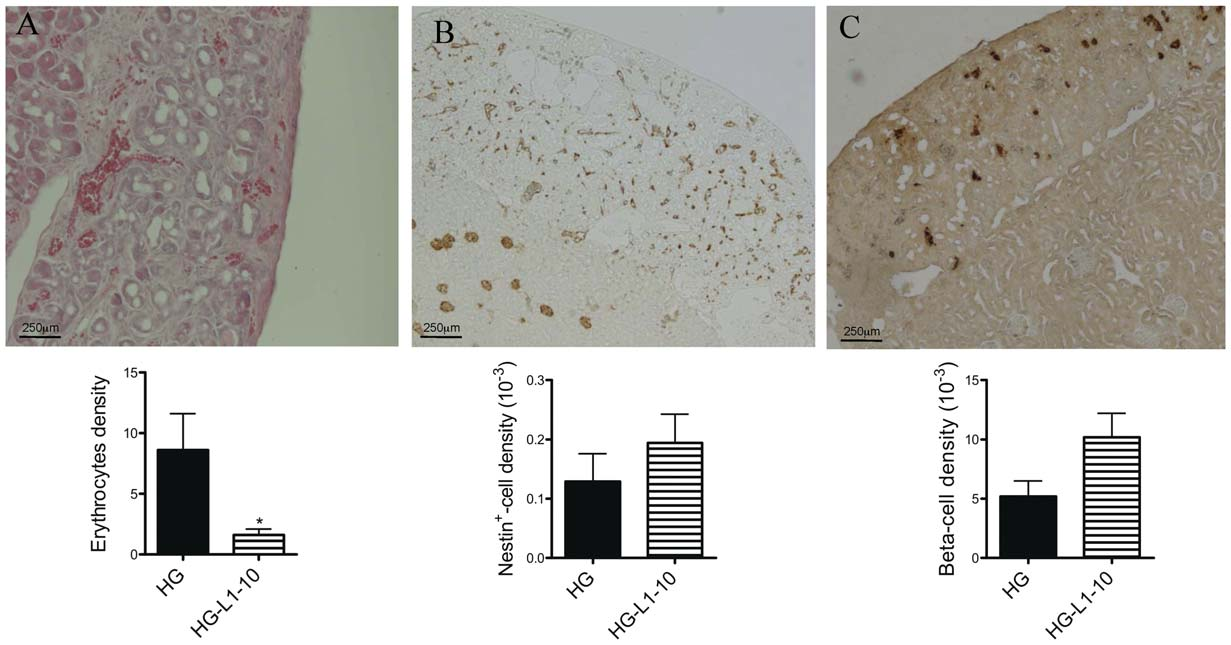
L1–10- induced specific Ang2 inhibition in hyperglycaemic conditions. During the 2 weeks post-graft, STZ-induced diabetic SCID mice received L1–10 (4 mg/kg, twice-a-week). Pancreata were then collected (A) and vascularization was analyzed by hematoxylin-eosin staining (B) and nestin immunohistochemistry (C). Large blood-filled spaces were decreased by L1–10 treatment (E). Insulin staining (G) showed a trend towards increased β-cell density by L1–10 treatment.

To explore this possibility, we treated SCID mice, twice-a-week during the 2 weeks following grafts, with 4 mg/kg i.p. of L1–10, an Ang-2 specific inhibitor [28], [29]. In normoglycaemic conditions, inhibition of Ang2 had no effect on graft development and vascularization (data not shown). By contrast, L1–10 treatment improved pancreatic vascularization in hyperglycaemic SCID mice (Fig. 6), based on the following facts: 1) L1–10 treatment decreased by 5-fold the large blood-filled pancreatic spaces of hyperglycaemic mice, as assessed by quantification of erythrocytes density per vessel (Fig. 6.A); 2) nestin-positive pancreatic endothelial cell numbers were increase in hyperglycaemic L1–10 treated mice (Fig. 6.B); 3) there were also less vessel discontinuities; 4) and finally, there was a concomitant, increase in beta-cell density in L1–10-treated hyperglycaemic SCID mice (Fig. 6.C).

## Discussion

Pancreatic epithelia and vessels have strong reciprocal influences [1]. To study their interactions during pancreas development in normo- and hyperglycaemic conditions, we grafted embryonic chick pancreata under SCID mouse kidney capsules. It is possible with this methodology to characterize the origin, avian (pancreatic) or murine (vascular), of the growth factors involved and to modulate them specifically. We observed during normoglycaemic conditions that the vessels penetrating the graft were from the host after characterization using both *in situ* hybridization with both mouse- and chick-specific VEGFR2 probes and immunohistochemistry for nestin, a specific mouse endothelial cell marker. Graft vascularization by recipient's vessels and endothelial cells has been previously demonstrated in particular in islets xenografts studies [30] and metanephroi transplantation experiments [31]. Second, we observed major vascular anomalies in the grafted pancreas of hyperglycaemic SCID mice, including endothelial discontinuities, large blood-filled spaces and decreased vessel density. We then studied the expression of pro- or anti-angiogenic factors potentially involved in this hyperglycaemia-induced vasculopathy. While expression of both VEGF and Tsp1 was not altered, that of Ang2 was strongly upregulated and may have been causal in vascular defects. In a second set of experiments, to improve vascular defects, we first over-expressed VEGF specifically in the graft, not in the whole diabetic mice, by using a RCAS expression vector. This failed to improve vascularization. We then focused on the anti-angiogenic factor Ang2. We treated mice with L1–10 a specific Ang2 inhibitor [29]. Ang2 inhibition corrected the vascular defects, suggesting that Ang2 may be involved in hyperglycaemia-induced pancreatic vasculopathy.

There are numerous similarities in vascular abnormalities presented by mice experiencing high Ang2 levels and those we observed in pancreatic grafts submitted to hyperglycaemia. Transgenic mice over-expressing Ang2 exhibit vessel discontinuities, with detachment of the endothelium from the underlying mesenchyme and widespread vessel discontinuities [32]. Chronic systemic Ang2 delivery to mice alters blood vessels anatomy both qualitatively and quantitatively [33]. Several studies have shown a link between Ang2 and diabetic vascular pathology. Ang2, and not Ang1, levels are elevated in plasma patients with type 2 diabetes and this increase correlates with myocardial damage [34], [35]. Circulating levels of Ang2 are significantly higher among diabetic treated with insulin, i.e. those who have the highest insulin secretory defects [36]. Expression of Ang2, and not that of Ang1, is also increased in the heart of STZ-hyperglycaemic mice with or without myocardial ischemia [37] and in db/db mice after myocardial ischemia [38]. As reviewed by Hammes et al., chronic hyperglycemia induces upregulation of Ang2 in retinal endothelial cells and Müller cells, leading to retinal pericyte detachment, migration, apoptosis, and progressive vasoregression [39]. Type 2 diabetic mice show increased Ang2 expression in the ischemic brain after stroke [40]. Finally, impaired wound healing is associated with increased Ang2 protein expression in db/db mice and STZ-hyperglycaemic rats [41], [42]. In these studies, Ang1 protein expression was not increased.

Several hypotheses can be made on the role of Ang2 in diabetic vasculopathy. First, Ang2 alone may exert pro-angiogenic activity [32]. Second, high Ang2 levels may destabilize vessel walls and consequently favor VEGF action and/or reduce the threshold level for VEGFR2 activation and signaling [33]. Indeed over-expression of VEGF itself has been associated with vascular abnormalities [43]. We blocked Ang2 action by using L1–10, an anti-Ang2 peptide-Fc fusion protein, which has been used to inhibit tumor development [28], [29]. L1–10 is a compound related to L1–7, which was used in previous studies and shown to be a specific inhibitor of Ang2 [29]. When measuring the neutralization of the angiopoietin:Tie2 interaction, L1–10 showed >1000-fold selectivity for Ang2 over Ang1. L1–10 was also recently shown to correct vascular abnormalities associated with Ang2 over-expression in transgenic mice over-expressing the forkhead box C2 in adipose tissue [44]. In our work, L1–10 improved pancreatic development, differentiation and vascularization in hyperglycaemic conditions, suggesting that Ang2 may have causal in our settings.

As type 2 diabetic patients exhibit increased levels of circulating Ang-2 [36], we hypothesized that Ang2 could impact on pancreatic vascularization during type 2 diabetes. Ang2, is expressed during normal pancreas development [45]. Ang2 is highly correlated with vascular inflammation in lupus [46], psoriasis [47] and rheumatoid arthritis [48]. Transplantation of islets of Langerhans is an emerging treatment procedure for patients with severe type 1 diabetes. After an initial avascular engraftment period [49], islets grafts become revascularized from both intra-islet and recipient-derived endothelial cells [30], [50]. Acquired vasculature has a lower vessel density compared with the endogenous islets [51]. In animal models, therapies that enhance the angiogenic capacity of islets by over-expression of VEGF-A, Ang1 increase the vascular density of islet grafts and improve metabolic function [3], [52]. When islets were pre-cultured for 7 days, they expressed Ang2 [53]. Glucotoxicity is regularly hypothesized to be a factor affecting the outcome of islet transplantation, acting through altered angiogenesis [54], [55]. Exposure to hyperglycemia at the beginning of the graft could alter the revascularization process and impact on islet survival and implication of Ang2 still need to be investigated.

This work highlighted an important role of Ang2 in pancreatic vascular defects induced by hyperglycaemia. As inhibition of Ang2 improved vascularization and beta-cell density, Ang2 contribution to other models of type 2 diabetes and in altered revascularization of grafted pancreatic islets in type 1 diabetes should be investigated.

## Methods

### Ethics Statement

This study was carried out along the principles of laboratory animal care, experiments were approved by INSERM (the French National Institute of Health) and was approved by the institutional research ethics committee (Animalhouse agreement number B 75- 05- 12; SC permit number B75-1571).

### Animals and pancreatic graft under the kidney capsule

Seven-week-old female SCID mice were obtained from Charles River (L'Arbresle, France) and kept in the animal house of College de France. One week after arrival, diabetes was induced by a single intraperitoneal injection (i.p.) of streptozotocin (STZ, Sigma Aldrich, Saint Quentin Fallavier, France) freshly dissolved in 0.1 mol/l citrate buffer at pH 4.5 and delivered at a dose of 160 mg/kg. Control SCID mice were injected with an equal volume (100 ml) of citrate buffer. Blood glucose concentration was measured using a Free style Papillon reflectance meter (Abbott, Rungis, France) on samples collected from the tail vein. Diabetes was defined as blood glucose levels consistently exceeding 200 mg/dl (Fig. 1). Mice were used for grafting experiments between 3 to 5 days after STZ or citrate buffer injections.

Fertilized White Leghorn chicken eggs were incubated at 37.9°C in a humidified atmosphere (>60% relative humidity) as previously described [26]. At the 14^th^ day postincubation (dpi), chick pancreas was microdissected and immediately grafted under the kidney capsule of SCID mice, which were anesthetized by a ketamine/xylazine mix (Imalgen 1000, 120 mg/kg, Merial, Lyon, France; Rompun, 6 mg/kg, Bayer Pharma, Puteaux, France). Mice were sacrificed 2 weeks later and the grafted kidney was collected for histology analyses. Blood glucose levels were followed every 2–3 days during these two weeks.

### Retroviral infection of embryonic chick pancreas with RCAS-VEGF

We used the plasmid construct VEGF expressed by the avian retrovirus vector RCAS (RCAS-VEGF) a gift from Peter Vogt [56]. RCAS retroviruses were produced from chick embryonic fibroblasts as previously described [57]. Embryonic chick-pancreata were infected *in vitro* during one hour with RCAS-VEGF, just before grafting.

### Histochemistry

Chick pancreas either before graft (at 14 dpi) or 2 weeks after graft (pancreas on kidney) were fixed for 1 and 4 h, respectively, in 4%-paraformaldehyde and processed for paraffin embedding. Each pancreatic block was serially sectioned (7 µm) throughout this length and was then mounted on slides.

### Immunohistochemistry

Endocrine and exocrine tissues were stained using guinea pig polyclonal anti-insulin (1/200; Dako, Glostrup, Denmark), rabbit polyclonal anti-glucagon (1/200; Dako) or rabbit polyclonal anti-alpha amylase (1/200; Sigma, Saint-Louis, Missouri, USA) primary antibodies. Then, we added biotinylated anti-guinea pig or anti-rabbit antibodies (1/200; Vector) as secondary antibodies, followed by the use of Vectastain ABC kit (Vector) and diaminobenzidine (DAB). Endothelial cells were immunostained with anti-nestin primary antibody (1/50; Santa Cruz Biotechnology, Santa Cruz, California, USA), after an antigen retrieval induced by microwave pretreatment in 10 mmol/L citrate buffer (pH 6). Slides were then incubated with the tyramide amplification signal (TSA kit, Perkin Elmer, Courtaboeuf, France) and staining was revealed using DAB. Nestin is a specific marker for pancreatic endothelial cells and, in particular, proliferative endothelial cells [19]. To observe erythrocytes, slides were stained with hematoxylin/eosin.

### Morphometry

Immunohistochemistry morphometry analyses were performed using iVison program (iVision-Mac, version 4.0.7, Biovision technologies, Exton, PA) on 6–8 sections per pancreas from 3–5 animals. Average size of pancreas was expressed in µm^2^. Beta-cell density was expressed as the ratio of average beta-cell surface on total pancreas surface, vascular density as the ratio of average nestin-stained vessel surface on pancreas total surface and erythrocyte density as ratio of average erythrocyte-filled vessel surface on total vascular nestin-stained compartment.

### In situ hybridization

In situ hybridization was performed as previously described on paraffin sections [58]. The antisense and sense probes used chicken (c)-Ang2, mouse (m)-Ang2, cVEGF, mVEGF, humanTsp1, cVEGFR2 and mVEGFR2 have been previously described [26], [28], [59], [60]. In situ hybridization analyses were assessed by semi-quantitative scoring and were expressed in arbitrary units (a.u.).

### Angiopoietin-2 blocking experiments

L1–10, an FC-fusion protein Ang2-specific inhibitor [29] was kindly provided by Amgen, (Thousand Oaks, CA). L1–10, 4 mg/kg in PBS was injected i.p. twice- a-week from the day of graft onwards to the day of sacrifice (14 dpi).
